# Nonalcoholic fatty liver disease and bariatric surgery: a comprehensive review: Doença hepática gordurosa não alcoólica e cirurgia bariátrica: uma revisão abrangente

**DOI:** 10.1590/1516-3180.2016.0306311216

**Published:** 2017-05-29

**Authors:** Everton Cazzo, José Carlos Pareja, Elinton Adami Chaim

**Affiliations:** I MD, MSc, PhD. Assistant Professor, Department of Surgery, Universidade Estadual de Campinas (UNICAMP), Campinas (SP), Brazil.; II MD, PhD. Associate Professor, Department of Surgery, Universidade Estadual de Campinas (UNICAMP), Campinas (SP), Brazil.; III MD, MSc, PhD. Full Professor, Department of Surgery, Universidade Estadual de Campinas (UNICAMP), Campinas, (SP), Brazil.

**Keywords:** Fatty liver, Obesity, Bariatric surgery, Metabolic syndrome X, Insulin resistance

## Abstract

**CONTEXT AND OBJECTIVE:**

Nonalcoholic fatty liver disease (NAFLD) has been increasingly diagnosed worldwide and is now recognized as a source of public health concern. It comprises a wide spectrum of histological features that range from simple steatosis to severe forms of fibrosis, steatohepatitis and even cirrhosis. The impact of bariatric surgery on the course of NAFLD in individuals with obesity has been extensively studied.

**DESIGN AND SETTING:**

Narrative review; public university hospital.

**METHODS:**

A comprehensive review was conducted based on an online search on the electronic databases MEDLINE and LILACS using the MeSH terms “fatty liver” and “bariatric surgery”.

**RESULTS:**

The exact mechanisms that lead to improvement in NAFLD following bariatric surgery are not completely understood. Since Roux-en-Y gastric bypass (RYGB) is the bariatric surgical procedure most performed worldwide, it is also the one from which the effects on NAFLD have been most studied, although there is also consistent evidence regarding the effects from gastric banding, sleeve gastrectomy and biliopancreatic diversions.

**CONCLUSION:**

According to the currently available evidence, bariatric surgery leads to significant improvement in NAFLD. Further research, especially by means of randomized controlled trials enrolling larger cohorts of individuals, is needed to determine the optimal procedure for this group of subjects.

## RESUMO

**CONTEXTO E OBJETIVO:**

A doença hepática gordurosa não alcoólica (DHGNA) tem sido diagnosticada com maior frequência em todo o mundo na atualidade, sendo agora reconhecida como motivo de preocupação em saúde pública. Abrange um amplo espectro de alterações histológicas que variam desde a simples esteatose até formas graves de fibrose, esteato-hepatite e até cirrose. O impacto da cirurgia bariátrica sobre o curso da DHGNA em indivíduos com obesidade tem sido profundamente estudado.

**TIPO DE ESTUDO E LOCAL:**

Revisão narrativa; hospital universitário público.

**MÉTODOS:**

Uma revisão abrangente da literatura foi conduzida baseada na pesquisa *on-line* nas bases de dados eletrônicas MEDLINE e LILACS por meio dos termos MeSH “fígado gorduroso” e “cirurgia bariátrica”.

**RESULTADOS:**

Os mecanismos exatos que levam à melhora da DHGNA após a cirurgia bariátrica não são completamente conhecidos. Como o *bypass* gástrico em Y de Roux é a cirurgia bariátrica mais realizada em todo o mundo, é também o procedimento cujos efeitos sobre DHGNA foram mais estudados, embora haja também evidências consistentes sobre os efeitos de banda gástrica, gastrectomia vertical e derivações biliopancreáticas.

**CONCLUSÃO:**

De acordo com as evidências atualmente disponíveis, a cirurgia bariátrica leva à melhora significativa da DHGNA. Mais estudos, especialmente por meio de ensaios clínicos randomizados e controlados, recrutando coortes maiores de indivíduos, são necessários para determinar o melhor procedimento para esse grupo de pacientes.

## INTRODUCTION

Nonalcoholic fatty liver disease (NAFLD) is defined as accumulation of excessive fat in the liver in the absence of excessive drinking of alcohol and/or any secondary cause.^1^^,^^2^^,^^3^ Along with obesity epidemics, it has been increasingly diagnosed over the last few decades and is now recognized as a source of public health concern. Today, NAFLD is considered to be the commonest liver disease worldwide. It comprises a wide spectrum of histological features that range from mild steatosis to severe forms of fibrosis, steatohepatitis and even cirrhosis.^3^^,^^4^

### Epidemiology

Today, the most reliable values for the prevalence of NAFLD and nonalcoholic steatohepatitis (NASH) in the general population in the Western world are 20-30% and 2-3%, respectively.^5^^,^^6^^,^^7^ In Asia, the prevalence of NAFLD has been found to range from 15% to 30% in the general population.^8^ The reported prevalence of NAFLD may vary according to the population studied and the diagnostic method used. The results from population-based studies provide more accurate data than do autopsies, hospital series or studies on high-risk groups, since they avoid higher selection bias. Studies that have detected NAFLD by means of liver enzyme levels have observed prevalences of NAFLD in the general population of between 3% and 23%, i.e. generally lower than those reported from analyses that used imaging methods.^5^^,^^6^^,^^7^^,^^8^^,^^9^^,^^10^^,^^11^ Studies based on ultrasound scans appear to be more reliable and have shown prevalences of NAFLD among healthy adults of 25-30% in the United States and Italy and 29% in Japan.^5^^,^^6^^,^^7^^,^^8^

NAFLD is associated with significantly higher overall mortality, compared with the general population, particularly due to cardiovascular and liver-related complications. The histological stage is nevertheless crucial for the longer-term prognosis. Among patients with NASH, compared with patients with simple steatosis, the prevalence of cirrhosis development within the first 15 years of follow-up is significantly higher (10.8% versus 0.7%, respectively) and liver-related mortality is also significantly higher (7.3% versus 0.9%). The overall and liver-related mortality among patients with NASH is correspondingly higher than would be expected among individuals of the same age and gender with simple NAFLD.^8^^,^^9^^,^^10^^,^^11^

Among obese populations with metabolic syndrome (MetS) and type 2 diabetes mellitus (T2DM), the prevalence of NAFLD is much higher, ranging from about 50% to 90%. NAFLD has become a challenging public health concern, given that the prevalences of obesity and overweight have increased to epidemic levels over the last few decades. It constitutes an additional risk factor for this group of patients, especially because of the possibility of evolution to severe forms of liver fibrosis (including end-stage liver disease and cirrhosis) and even liver cancer.^5^^,^^6^^,^^7^^,^^8^^,^^12^

Among cases labelled as “cryptogenic cirrhosis”, the prevalence of NAFLD appears to be very high. Nayak et al.^9^ carried out a study in which the clinicopathological features of NAFLD were explored through clinical data and by examining the explanted livers of living-donor liver transplant recipients. Among 103 adult liver transplant recipients with different types of chronic liver disease, 30 had a pre-liver transplantation diagnosis of cryptogenic cirrhosis. Out of these 30 cryptogenic cirrhosis cases, 19 (63.3 %) were finally labeled as NAFLD-related cirrhosis and showed histological features that differed in several respects from those reported for the early and established phases of NAFLD.

The risk factors for development of NAFLD have been established. Increasing age presents a direct correlation with the prevalence of NAFLD. This is possibly related to the increasing insulin resistance and incidence of metabolic syndrome that occurs with age.^12^ NAFLD generally occurs at a higher rate among men than among premenopausal women. It also differs according to ethnicity, such that it affects 45% of Hispanic people, 33% of Caucasians and 24% of African-Americans in the United States and has been recorded at higher levels among South Asians in the United Kingdom.^7^^,^^8^^,^^9^^,^^10^^,^^11^^,^^12^ Likewise, among children and adolescents, the prevalence of NAFLD again seems to differ between different ethnic groups, with the highest prevalence in Hispanic people.^5^

Several studies have revealed high rates of NAFLD among obese populations who undergo bariatric surgery.^13^^,^^14^^,^^15^^,^^16^^,^^17^ Reports on morbidly obese populations have shown that the prevalence of liver fibrosis ranges from 6% to 74.4% and steatohepatitis from 26% to 55%.^16^^,^^17^

### Pathophysiology

The underlying mechanisms that are present in the development of NAFLD are not completely understood, although much progress has been made over recent years. The classical pathogenetic pathway that leads to NAFLD was described as the “two-hit hypothesis”. This theory stated that damage to the liver tissue begins through triglyceride accumulation (the first hit), which functions as a sensitizing factor for secondary damage caused by inflammatory mediators (mainly cytokines or adipokines), mitochondrial dysfunction and oxidative stress (the second hits). These secondary aggressions would lead to steatohepatitis and fibrosis.^18^^,^^19^ Today, it is widely recognized that free fatty acids (FFAs) also play a direct role in promoting liver injury. One of the consequences of insulin resistance (IR) is greater influx of FFAs to the liver: these undergo β-oxidation or esterification with glycerol to form triglycerides. Additionally, FFAs are able to cause toxicity through directly increasing oxidative stress and activating inflammatory pathways.^20^^,^^21^ Another “hit” that was recently identified consists of impaired hepatocyte proliferation. In NAFLD, when cell death occurs in the liver tissue, oxidative stress causes disruption to the regenerative process, thus leading to proliferation of hepatic progenitor (oval) cells. The size of the oval cell population correlates directly with the degree of fibrosis and it is believed that activation of these cells is involved in hepatic carcinogenesis.^18^^,^^21^^,^^22^^,^^23^

In NAFLD, lipid accumulation within the liver (steatosis) may result from several mechanisms: increased fat synthesis, increased fat delivery, decreased fat export and/or decreased fat oxidation.^23^^,^^24^^,^^25^^,^^26^^,^^27^^,^^28^

IR plays an important role in NAFLD development and evolution, and NAFLD also contributes towards worsening of IR. In individuals with IR who present excess visceral fat, the influx of FFAs to the liver is increased, thus leading to both triglyceride accumulation and direct lipotoxicity. On the other hand, several abnormalities intrinsic to NAFLD impair insulin receptor signaling, especially FFAs, tumor necrosis factor-alpha (TNF-α), nuclear factor kappa B (NF-κB), ceramide, Jun N-terminal kinase 1 (JNK1), suppressors of cytokine signaling (SOCS) and cytochrome CYP2E1. The cumulative effect generates a vicious cycle of IR and NAFLD worsening each other.^18^^,^^23^^,^^29^^,^^30^^,^^31^

Steatosis is strongly associated with chronic hepatic inflammation, an effect that is partly mediated by activation of the Iκκ-β/NF-κB signaling pathway. This is associated with elevated hepatic expression of inflammatory cytokines such as TNF-α, interleukin-6 (IL-6) and interleukin 1-beta (IL-1β), and activation of Kupffer cells. Both serum and hepatic levels of TNF-α are elevated in patients with NASH, and these levels are correlated with histological severity.^18^^,^^32^^,^^33^^,^^34^^,^^35^ In addition to the proinflammatory effect, TNF-α promotes IR. Further data suggests that inflammation and NF-κB activation may also promote carcinogenesis and that this chronic inflammatory state may also play a key role in hepatocellular carcinoma (HCC) development.^18^^,^^36^^,^^37^

Increased leptin levels are observed in cases of obesity and NAFLD. These are usually regarded as states of leptin resistance. It is possible that leptin may have an important role in the pathogenesis of NAFLD. Conversely, the circulating levels of adiponectin are inversely proportional to body fat content and become reduced in patients with NAFLD.^38^^,^^39^^,^^40^^,^^41^

Oxidative stress and mitochondrial dysfunction play a well-reported role in NASH, with a direct correlation between the degrees of oxidative stress and the severity of liver disease. Structural mitochondrial abnormalities and impairment of mitochondrial respiratory chain activity have been reported in human studies.^18^^,^^42^^,^^43^^,^^44^^,^^45^^,^^46^

Endoplasmic reticulum (ER) stress and endotoxemia derived from the gut microbiota are other mechanisms that appear to be implicated in NASH pathogenesis. ER stress is associated with various types of biological stress, such as hyperinsulinemia and hyperlipidemia, and it usually causes IR, inflammation, apoptosis and mitochondrial dysfunction, through several mechanisms. In cases of alcohol-related steatohepatitis, ER stress is a well-established pathophysiological factor, but further study on the possible role that it plays in the development of NASH is needed.^18^

The role of gut bacterial overgrowth in the pathogenesis of NASH is now emerging through recent evidence. Bacterial overgrowth leads to production of ethanol and release of bacterial lipopolysaccharides, both of which can activate TNF-α production in Kupffer cells, thereby inducing inflammation in liver tissue. Individuals with NASH more frequently present bacterial overgrowth in the small intestine and increased gut permeability than do controls. These findings also suggest that this is a possible explanation for the rapid onset of NASH and liver fibrosis following jejunoileal bypass surgery. Furthermore, there is evidence that hepatic inflammation can be reduced by means of changes to the gut microbiota induced by antibiotics and probiotics.^47^^,^^48^^,^^49^^,^^50^^,^^51^^,^^52^^,^^53^^,^^54^^,^^55^^,^^56^

Under high levels of oxidative stress, which is a feature usually observed in the presence of injurious factors such as NASH and viral infection, mature hepatocytes may present impairment of their replication process, thus leading to reduction of their proliferative ability. Subsequently, hepatic progenitor cells (HPCs) are recruited; these cells have the capacity to differentiate into either hepatocytes or cholangiocytes and may be involved in carcinogenesis relating both to hepatocellular carcinoma and to cholangiocarcinoma. Initial research has observed that there is a strong association between expansion of HPCs and a ductular reaction in liver biopsy specimens in NASH cases. The extent of ductular reactions in turn strongly correlates with the degree of fibrosis, thus suggesting that HPC expansion/ductular reaction may play an important role in stimulating progressive periportal fibrosis.^57^^,^^58^^,^^59^^,^^60^

### Histopathology

NAFLD encompasses a wide spectrum of liver histopathological abnormalities that may also have highly variable natural courses. It is grossly divided into two main categories: simple steatosis, in which only hepatocellular steatosis is observed; and NASH, in which necroinflammatory reactions are associated with hepato-cellular steatosis. Simple steatosis usually presents a benign course. NASH, on the other hand, is a progressive disease that can evolve into liver cirrhosis and HCC. Steatohepatitis-associated hepatocellular carcinoma (SH-HCC) is a variant of classical HCC in which the onset appears to be strongly linked to NASH.^61^^,^^62^

It is important to emphasize that NAFLD/NASH presents a different pattern in pediatric individuals, with lesions that may or may not resemble those observed in adults. It constitutes an area of ongoing investigation within pathology.^61^^,^^62^ Since pediatric NAFLD is not the focus of this review, it will not be tackled here.

### Histopathological features of adult NAFLD

Hepatocellular steatosis is the hallmark of NAFLD; steatosis in more than 5% of the hepatocytes is required for the diagnosis of NAFLD.^61^^,^^63^^,^^64^ Steatosis may be macrovesicular or microvesicular; in NAFLD, the macrovesicular type is usually present, although about 10% of the cases may also present microvesicular steatosis. It usually begins in zone 3 and may evolve to panacinar steatosis in severe cases.^61^^,^^65^^,^^66^

Mild intralobular inflammation is present in NASH and consists of an infiltrate of mixed inflammatory cell types (lymphocytes, neutrophils, eosinophils and Kupffer cells). Satellitosis may be sporadically observed in NASH and consists of ballooned hepatocytes surrounded by polymorphs; this feature is more commonly associated with alcoholic hepatitis. Lipogranulomas and lobular microgranulomas are frequently seen in NASH. Portal inflammation in NASH is usually absent or mild; when there is disproportionately severe portal inflammation, the possibility of other concurrent hepatic diseases needs to be considered.^61^^,^^67^^,^^68^

Hepatocellular ballooning is another feature associated with NAFLD and is characterized by the presence of swollen hepatocytes with rarefied cytoplasm, which may contain fat droplets and Mallory-Denk bodies. It usually reflects hepatocellular injury.^61^

Fibrosis in NASH characteristically follows a perisinusoidal/pericellular pattern and usually begins in zone 3. Typically, an active necroinflammatory reaction is observed, along with fibrosis. Progression of perisinusoidal/pericellular fibrosis may lead to occurrences of portal/periportal fibrosis, bridging fibrosis and liver cirrhosis.^69^^,^^70^

Other important features of NAFLD/NASH include glycogenated nuclei, acidophil bodies, Mallory-Denk bodies (MDBs), iron deposition and megamitochondria.^61^^,^^62^ Glycogenated nuclei are vacuolated nuclei that are usually observed in periportal hepatocytes. They are common in NAFLD, but only rarely observed in alcohol-induced liver disease.^71^ Presence of apoptotic hepatocytes (acidophil bodies), usually in the sinusoids, is also commonly observed in NASH.^72^ The eosinophilic irregular-shaped aggregates that are usually observed in the cytoplasm of ballooned hepatocytes in zone 3 are known as MDBs. They are not specific to NASH and may be found in alcohol-induced disease, chronic cholestasis and HCC.^73^^,^^74^ Mild iron deposition within hepatocytes or in the sinusoidal lining cells of the reticuloendothelial system, or in both of these, is common in NAFLD/NASH; its significance is yet to be fully understood.^75^ Megamitochondria are massively enlarged round or crystal-shaped eosinophilic structures that are observed in the cytoplasm of hepatocytes, usually with concurrent microvesicular steatosis; they are believed to originate due to lipid peroxidation.^76^ Ductular reactions and presence of an arterial branch in zone 3 within perisinusoidal fibrosis are other poorly understood abnormalities observed in NASH.^59^

### Diagnosis

According to the current guidelines of the American Association for the Study of Liver Diseases (AASLD), there are four requirements for diagnosing NAFLD:

1. presence of hepatic steatosis detected using imaging methods or biopsy;
2. absence of significant alcohol consumption;
3. absence of possible competing etiologies for hepatic steatosis;
4. absence of concurrent chronic liver disease.^77^

There are several noninvasive methods for assessing and diagnosing NAFLD, although none of them are able to provide the same amount of information that is brought through liver biopsy.^78^ Ultrasound scanning is a valuable imaging tool, since it is cheap and highly available and may provide overall accuracy of nearly 80% for detecting NAFLD. On the other hand, it presents limitations that may compromise interpretation of the results, given that it is examiner-dependent and there may be technical difficulties in performing it on obese individuals because of their anatomical features. Thus, although ultrasound provides useful clues towards diagnosing NAFLD, it cannot be taken to be the ultimate method for detecting it. Moreover, it cannot provide nuanced evaluation of the presence of steatohepatitis and the severity of fibrosis.^17^^,^^79^^,^^80^

Ultrasonography-based transient elastography (FibroScan) is a promising method that can bring reliable results by means of safe examination. However, its cost and low availability compromise its potential impact.^17^^,^^81^ Furthermore, FibroScan has no value for patients with ascites and presents great limitations relating to examining obese patients, because the probe is calibrated for a specific distance between the liver and the chest wall and the low-frequency vibration induced by the probe and/or the ultrasound wave may be strongly attenuated by the fatty tissue.^82^^,^^83^^,^^84^

Development of noninvasive scores for assessing liver disease, including NAFLD, is an important field of study nowadays. The ultimate goal in using these scores is to obtain reliable information from noninvasive laboratory and clinical variables that are available in general practice. The NAFLD fibrosis score is the one most used today and can easily be calculated based on six readily available variables: age, body mass index (BMI), hyperglycemia, platelet count, albumin and aspartate transaminase/alanine transaminase (AST/ALT) ratio. A meta-analysis on 13 studies that enrolled 3,064 patients showed that the NAFLD fibrosis score yielded an area under the receiver operating characteristic curve (AUROC) of 0.85 for predicting advanced fibrosis (bridging fibrosis or cirrhosis). Scores ≤ -1.455 had 90% sensitivity and 60% specificity for ruling out advanced fibrosis, whereas scores > 0.676 had 67% sensitivity and 97% specificity for identifying the presence of advanced fibrosis.^85^ However, this score does not replace liver biopsy and cannot in any manner be regarded as a gold standard, given that it does not provide a nuanced evaluation. On the other hand, it does not produce any morbidity and can be easily and readily assessed through routine studies. Thus, this score is quite adequate for population-based studies and for clinical screening and follow-up purposes.^17^^,^^84^^,^^85^

Despite recent developments in noninvasive methods for assessing NAFLD, liver biopsy remains the gold-standard method for diagnosing and characterizing it, since it may provide a nuanced evaluation that no other method is capable of today. On the other hand, it is expensive and highly invasive, and carries significant risk of morbidity and even mortality.^77^ Moreover, the histological features of NAFLD and NASH are unevenly distributed throughout the entire liver parenchyma, and sampling error might result in substantial misdiagnosis and staging inaccuracies.^61^^,^^78^ The current guidelines state that use of biopsies should be restricted to individuals who would benefit the most from diagnostic and therapeutic guidance, and from prognostic perspectives. Hence, a biopsy should be considered for NAFLD assessment among patients:

1. with NAFLD, who are at increased risk of development of fibrosis and steatohepatitis;
2. with suspected NAFLD, in whom concurrent etiologies for hepatic steatosis and/or coexisting chronic liver diseases cannot otherwise be ruled out.^77^

### Current clinical treatment

The current treatment strategies for NAFLD are based on the various aspects of its pathophysiology, especially its relationship with obesity and insulin resistance. The current nonsurgical strategies can be divided in non-pharmacological and pharmacological.

The primary goals of the main nonpharmacological strategies are weight loss and lifestyle changes. Interventions based on low-fat and low-calorie diets have been evaluated in several studies in which it was observed that these diets were associated with reductions in liver enzyme levels and liver fat content and improvement in liver histology.^87^^,^^88^^,^^89^^,^^90^^,^^91^^,^^92^^,^^93^ Thoma et al. reviewed the efficacy of physical exercise alone versus physical exercise combined with dietary approaches to reduce liver fat content. Energy restriction and weight reduction of 4-14% resulted in significant decreases in hepatic steatosis of 35-81%. Decreases in liver fat correlated most strongly with the degree of weight loss. It was seen that regular exercise might modestly reduce steatosis even without a change in weight.^94^ However, the greatest caveat of interventions that use dietary measures for weight loss is their lack of long-term durability. It remains unclear how weight regain affects the natural history of NAFLD/NASH.^94^^,^^95^^,^^96^^,^^97^

The primary goal of pharmacological interventions aimed towards NAFLD may either be weight loss or consist of a direct effect on liver disease and its pathophysiological features, especially insulin resistance and inflammation. Evidence regarding the effect of weight loss medications on NAFLD is scarce. The results relating to orlistat were inconclusive in several trials.^87^^,^^98^ Pentoxifylline, albeit not targeted for weight loss, led to a slight weight reduction and improvement in NASH in one trial.^99^

Regarding insulin resistance, the potential therapeutic effect of insulin sensitizers on NAFLD/NASH has gathered much attention. Rosiglitazone was shown to improve steatosis and aminotransferase levels in patients with NASH in a randomized controlled trial,^100^ but its use is prohibited in Europe and very restricted in the United States because it may increase the risk of ischemic heart disease.^101^ In randomized controlled trials, pioglitazone induced significant improvements in serum aminotransferase levels and liver histology (steatosis, inflammation, ballooning and Mallory-Denk bodies) in individuals with NASH.^102^^,^^103^^,^^104^ However, the improvement in the extent of fibrosis was not significant. Use of pioglitazone is also prohibited in Europe, due to the risk of bladder cancer.^101^ Use of metformin was not shown to provide any benefits or to have any independent therapeutic role in individuals with NAFLD, in a meta-analysis study. However, there is level III evidence that metformin may have a chemopreventive role in patients with diabetes and chronic liver disease, with reductions in the incidence of HCC and cholangiocarcinoma.^105^ Liraglutide, an analogue of glucagon-like peptide 1 (GLP-1), was shown to promote significant improvement in liver histology in individuals with biopsy-proven NASH after 48 hours of therapy. Longer-term research is needed, but the initial results appear promising.^106^

Use of vitamin E, because of its antioxidant effects, has shown benefits regarding liver histology among non-diabetic individuals with NASH. Nonetheless, it is important to emphasize that the safety of long-term usage of vitamin E is questionable, since high-dosage vitamin E supplements are associated with increased incidence of prostate cancer in healthy men, and with all-cause mortality.^101^^,^^107^^,^^108^^,^^109^^,^^110^

The current usage of lipid-lowering drugs targeted towards NAFLD (mainly statins) is so far unsupported by any substantial results from well-conducted studies. Thus, this practice is not recommended by American guidelines.^77^^,^^101^ The newer agent ezetimibe, which acts through inhibiting cholesterol absorption, has shown promising effects, especially in association with acarbose, but further results are needed in order to enable its use to be validated as a current recommendation.^111^^,^^112^^,^^113^^,^^114^

There is a lack of conclusive evidence supporting the use of ursodeoxycholic acid, N-3 polyunsaturated fatty acids, angiotensin receptor blockers, probiotics and synbiotics as validated treatment options for NAFLD.^77^^,^^101^

## OBJECTIVE

The aim of this study was to conduct a critical analysis on the evidence available regarding the effect of bariatric surgery on NAFLD.

## METHODS

A review of the literature was conducted through an online search for the MeSH terms “fatty liver” and “bariatric surgery” in MEDLINE (via PubMed) and LILACS (via BVS) (Table 1). We included original studies that reported on clinical trials on the effects of several types of bariatric surgery on NAFLD. All the papers were checked according to their titles and abstracts (screening). Full papers were obtained from journals available through the website of the Commission for Improvement of Higher Education Personnel (Comissão de Aperfeiçoamento de Pessoal de Nível Superior, CAPES) (Ministry of Education, Brazil). Unavailable articles were requested from their authors. Articles presenting potentially relevant studies were read and analyzed to assess their inclusion criteria. We excluded articles that consisted of *in vitro* or animal studies, articles in which the participants’ characteristics did not match those mentioned above, poster session abstracts, review articles and other types of publications (non-standard bariatric surgical techniques; studies without appropriate follow-up; studies without appropriate NAFLD assessment; or studies with critical methodological issues). Other papers were used for contextualization and discussion.

**Table 1.**
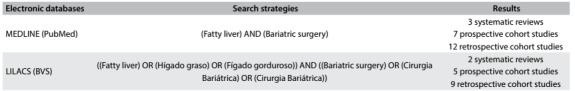
Database search results for the effects of bariatric surgery among individuals with nonalcoholic fatty liver disease, on November 20, 2016

## RESULTS

There was significant overlap between the databases. After careful analysis, we selected three systematic reviews, seven prospective cohort studies and twelve retrospective cohort studies. Table 2 summarizes the main articles found and their respective characteristics and reported results.^115^^,^^116^^,^^117^^,^^118^^,^^119^^,^^120^^,^^121^^,^^122^^,^^123^^,^^124^^,^^125^^,^^126^^,^^127^^,^^128^^,^^129^^,^^130^^,^^131^^,^^132^^,^^133^

**Table 2.**
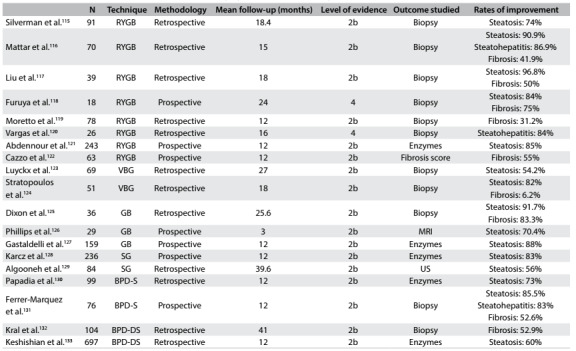
Main studies on the influence of bariatric surgery on nonalcoholic fatty liver disease

N = number of individuals; RYGB = Roux-en-Y gastric bypass; VBG = vertical banded gastroplasty; GB = gastric banding; SG = sleeve gastrectomy; BPD-S = biliopancreatic diversion - Scopinaro; BPD-DS = biliopancreatic diversion - duodenal switch; MRI = magnetic resonance imaging US = ultrasound scan. Levels of evidence according to the Oxford classification - 1a: systematic reviews (with homogeneity) of randomized controlled trials; 1b: individual randomized controlled trials (with narrow confidence interval); 1c: all or no randomized controlled trials; 2a: systematic reviews (with homogeneity) of cohort studies; 2b: individual cohort study or low-quality randomized controlled trials (e.g. < 80% follow-up); 2c: “outcomes” research; ecological studies; 3a: systematic review (with homogeneity) of case-control studies; 3b: individual case-control study; 4: case series (and poor quality cohort and case-control studies); 5: expert opinion without explicit critical appraisal, or based on physiology, bench research or “first principles”.

**Figure 1.**
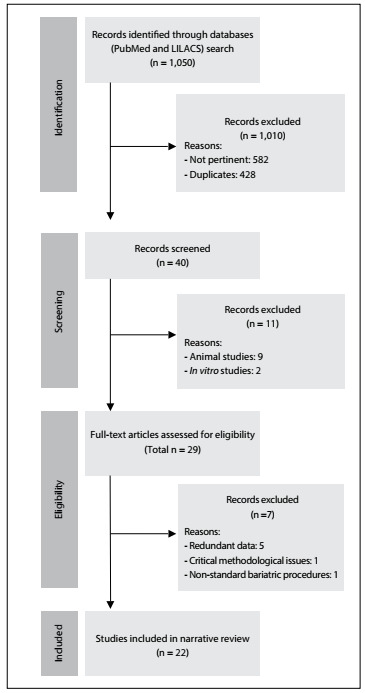
Flow diagram of the narrative review of the literature.

A schematic representation of the search in the online databases and the identification, exclusion and selection of the articles is presented in Figure 1.

### Bariatric surgery and NAFLD

The impact of bariatric surgery on the course of NAFLD in obese individuals has been extensively reported. Surgery plays a significant role in the expected natural history of NAFLD and usually leads to rapid changes in its evolution. Different surgical methods act within this context through a variety of mechanisms, which over the long term may be directly associated with the weight loss achieved, but over the short to medium term appear to be more related to acute structural and endocrine changes that are attributable to the procedures. Table 1 summarizes the main studies on the influence of bariatric surgery on NAFLD.

#### Roux-en-Y gastric bypass

A pioneering study conducted by Silverman et al.,^115^ which enrolled 91 individuals, revealed that after Roux-en-Y gastric bypass (RYGB), 65 patients presented reduced steatosis, 18 patients had no steatosis, 5 patients with minimal steatosis showed no change and 3 patients presented increased steatosis. Pre-gastric bypass biopsies from 13 patients showed perisinusoidal fibrosis (PSF) that was major with bridging in three patients, moderate in one patient and slight in nine patients. Following RYGB, PSF was found to have been eliminated in 10 patients, was reduced in one patient and was the same in two patients. One patient developed PSF after gastric bypass.

In a study that evaluated 70 individuals who underwent a variety of bariatric procedures by means of intraoperative and postoperative liver biopsies, Mattar et al.^116^ observed significant improvement in liver steatosis (from 88% to 8%), inflammation (from 23% to 2%) and fibrosis (from 31% to 13%). Inflammation and fibrosis resolved in 37% and 20% of the patients, respectively, corresponding to significant improvements of 82% in grade and 39% in the stage of liver disease. As expected, purely restrictive procedures produced a significantly less dramatic impact on weight loss than did gastric bypass. Improvement in grade in the purely restrictive group (66% showed improvement in grade) was significantly less than in the gastric bypass group (93%).

In a case series that enrolled 16 individuals, Clark et al.^134^ evaluated liver histology at the time of RYGB surgery and at the time of elective incisional hernia repair after weight loss. For all the patients, the initial biopsy showed steatosis, while 94% had inflammation, 88% had ballooning degeneration, 88% had perisinusoidal fibrosis and 81% had portal fibrosis. Steatosis improved in 15 out of the 16 patients, with resolution in 13. Twelve of the 15 patients with inflammation at the baseline showed improvement, and 12 out of 14 showed less ballooning. Six of the 14 patients with perisinusoidal fibrosis and 6 of the 13 patients with portal fibrosis showed improvement. No patient presented worsening of steatosis, inflammation, ballooning or fibrosis.

In a historical cohort that enrolled 90 individuals who underwent liver biopsies during RYGB and one year afterwards, Mottin et al.^135^ observed that the prevalence of hepatic steatosis at the time of the surgery was 87.6%. In the second biopsy, 16 patients (17.8%) out of the total had the same degree of steatosis, 25 (27.8%) presented an improved steatosis pattern and 49 (54.4%) had normal hepatic tissue.

In a study on 19 individuals who underwent liver biopsy during RYGB that confirmed the presence of NASH and were then contacted to undergo a repeat biopsy, Barker et al.^136^ observed significant improvements in steatosis, lobular inflammation, portal and lobular fibrosis. Histopathological criteria for NASH were no longer found in 17/19 patients (89%).

In a study that enrolled 16 individuals who underwent liver biopsy during primary RYGB and a new biopsy during repairs on incisional hernias, Csendes et al.^137^ observed that 11 out of 15 patients who had had liver abnormalities returned to a normal condition or only had minimal change (73.3%); two patients (13.3%) showed improvements, one patient presented a slight worsening of liver condition and one patient who had presented liver cirrhosis showed no change.

de Almeida et al.^138^ studied 16 individuals who underwent a first biopsy during RYGB and another subsequent to the surgery (23.5 ± 8.4 months afterwards). They observed complete regression of NAFLD in 15 patients (93.7%); complete regression of necroinflammatory activity was observed in all patients. Among the four patients presenting fibrosis in the first biopsy, complete remission was observed in one and improvement in one; two continued to show the same degree of fibrosis without evidence of disease activity.

An exploratory study carried out by Klein et al.^139^ evaluated whole-body glucose, fatty acid and lipoprotein kinetics, liver histology and hepatic cellular factors involved in inflammation and fibrogenesis in seven severely obese subjects, before and one year after RYGB. A decrease in hepatic steatosis was observed, without changes in standard histological assessments of inflammation and fibrosis. However, there were marked decreases in hepatic factors involved in regulating fibrogenesis (collagen-alpha 1, transforming growth factor-beta 1, alpha-smooth muscle actin and tissue inhibitor of metalloproteinase 1 expression and alpha-smooth muscle actin content) and inflammation (macrophage chemoattractant protein 1 and interleukin 8 expression).

In a retrospective analysis on paired needle liver biopsies taken during and following RYGB in 39 patients, Liu et al.^117^ observed that the initial prevalences of hepatic pathological conditions were: steatosis (89.7%), hepatocellular ballooning (58.9%) and centrilobular/perisinusoidal fibrosis (50%). These improved significantly after RYGB: steatosis (2.9%), ballooning (0%) and centrilobular fibrosis (25%); significant decreases in the lobular inflammation score and stage of fibrosis were also noted. Nonetheless, no improvements were seen in portal tract inflammation or fibrosis.

A controlled study conducted by Furuya et al.,^118^ which enrolled 18 individuals who underwent a wedge biopsy during RYGB and a percutaneous biopsy two years later, showed that, after a mean excess weight loss of 60%, steatosis disappeared in 84% of the patients, fibrosis in 75% and ballooning in 50%. A slight lobular inflammatory infiltrate remained in 81%, apparently unrelated to fatty degeneration.

Kakizaki et al.^140^ conducted a prospective study that enrolled 84 participants, to compare the effects of RYGB on NAFLD in Japanese and non-Japanese individuals. They observed that when the body mass index (BMI) was similar, liver dysfunction among Japanese patients with severe obesity tended to be higher than among non-Japanese patients. Japanese patients with severe obesity would therefore need to reduce their body weight to a greater degree than would non-Japanese patients with the same BMI. The laboratory data and BMI were seen to be significantly improved, one year after laparoscopic RYGB, in both groups.

Tai et al.^141^ conducted a prospective study that enrolled 21 patients with morbid obesity who underwent intraoperative liver biopsy and follow-up liver biopsy one year after laparoscopic RYGB. They observed statistically significant histological improvements in the patients’ NAFLD activity scores (NAS) and individual components, including steatosis, ballooning degeneration and lobular inflammation. Preoperatively, 4 (19.0%), 11 (52.4%) and 6 (28.6%) patients were found to have NAS ≥ 5, 3 to 4, and ≤ 2, respectively; all patients had NAS ≤ 2 after surgery. The stage of fibrosis also presented significant improvement.

A retrospective study by Moretto et al.^119^ that evaluated 78 patients with morbid obesity who underwent RYGB and had liver biopsies taken during surgery and after weight loss, observed that 35 (44.8%) had fibrosis at the time of the first biopsy and 24 (30.8%) had hepatic fibrosis after weight loss, including 19 of the 35 patients (54.3%) with fibrosis at the first biopsy and 5 of the 43 (11.6%) without hepatic fibrosis at the first biopsy. These authors concluded that surgical weight loss among patients with morbid obesity was associated with a significant reduction in the prevalence of hepatic fibrosis.

In a study on 26 paired biopsies collected during RYGB and at an average of 16 months afterwards, Vargas et al.^120^ reported that there were significant improvements in steatosis, lobular and portal inflammation and fibrosis in the second biopsy. Twenty-five of the patients (96.1%) presented NASH in their index biopsy, while only four (15.3%) of the repeat biopsies fulfilled the criteria for NASH. Persistence of fibrosis was observed in five patients at the second biopsy. Steatosis and fibrosis at surgery were predictors of significant fibrosis post-surgery.

In a large prospective study that enrolled 1236 participants, Caiazzo et al.^142^ compared liver biopsy outcomes following RYGB and gastric banding. All NAFLD parameters improved after surgery. They all improved significantly more after RYGB than after gastric banding (percentage steatosis: one year, 7.9 versus 17.9; five years, 8.7 versus 14.5; NAS: one year, 0.7 versus 1.1; five years, 0.7 versus 1.0). In multivariate analysis, the superiority of RYGB was primarily but not completely explained by weight loss.

Abdennour et al.^121^ conducted a prospective study that enrolled 243 individuals, with the main objective of studying the relationship between white adipose tissue evaluation methods and weight loss. These authors also observed significant improvement in liver enzymes one year after RYGB.

Cazzo et al.^122^ conducted a prospective study in which they evaluated changes in NAFLD fibrosis score between the time of surgery and one year after RYGB. They observed that the mean score decreased from 1.142 to 0.066, and that surgery led to a resolution rate for advanced fibrosis of 55%. Resolution was statistically associated with female gender, the percentage of excess weight loss, post-surgical BMI, postsurgical platelet count and diabetes resolution.

In an exploratory study that compared 14 RYGB and 9 sleeve gastrectomy (SG) patients, Froylich et al.^143^ observed that all morphological characteristics of NAS improved significantly after RYGB, whereas only steatosis and total NAS improved after SG. The state of fibrosis improved in both groups, but to a greater degree after RYGB. There were no significant differences in the decreases in NAS score after RYGB and SG procedures. These exploratory data support the idea that a randomized trial should be conducted to determine the differential effects of SG and RYGB on NAFLD.

#### Gastric banding and banded gastroplasty

Luyckx et al.^123^ conducted a retrospective clinical analysis on 69 individuals who underwent liver biopsy during gastroplasty and a repeat biopsy later on, during the course of a mandatory surgical procedure. They observed that, after the drastic weight reduction, 45% of the histological observations were considered normal. Abnormal fattiness significantly decreased following surgery (38% versus 83% before). Furthermore, the severity of steatosis was significantly reduced in most cases: mild in 62% versus 21%, moderate in 23% versus 37% and severe in 15% versus 42%, after and before weight loss, respectively. However, a significant increase in hepatitis was observed, in 26% versus 14% before. Nevertheless, 87.5% of the cases were graded as mild and 12.5% as moderate, while no severe hepatitis was observed. The prevalences of fibrosis and cirrhosis remained unchanged.

Busetto et al.^144^ examined a case series in which they aimed to evaluate visceral fat content following gastric banding. They enrolled six premenopausal women with morbid obesity with an ultrasonographic diagnosis of liver steatosis and observed that there was a statistically significant reduction in visceral adipose tissue, of 1.0 ± 0.9 liters over the period from 0 to 8 weeks, but only a non-significant further reduction of 0.6 ± 0.7 liters over the period from 8 to 24 weeks. The relative reduction of visceral fat over the period from 0 to 8 weeks was greater than the relative reduction of total fat. The liver volume also showed a statistically significant reduction of 0.24 ± 0.26 liters during the first phase of weight loss, corresponding to a relative reduction of 12.3 ± 10.6%. Hence, during the period from 8 to 24 weeks, liver volume was substantially stable. During the phase of rapid weight loss after gastric surgery, preferential mobilization of visceral fat, compared with total adipose tissue, was observed.

Stratopoulos et al.^124^ conducted a study that enrolled 51 individuals who underwent a biopsy during surgery and a second biopsy after an average time of 18 months, and among whom 16 underwent a third biopsy at an average of 17 months after the second one. They reported that, at baseline, steatosis and steatohepatitis (mostly grade 3) were present in 98.0% of the subjects and fibrosis (mostly stage 2) in 94.1%. After an excess weight loss of 66%, steatosis and steatohepatitis improved significantly. Although a significant overall decrease in fibrosis occurred, 21 patients (41.1%) did not present any change, while six patients (11.7%) showed increased fibrosis. None of the patients developed cirrhosis. The third biopsy, performed in 16 of the subjects, showed further significant improvements in liver histology.

Dixon et al.^125^ examined a case series of 36 selected obese patients and evaluated paired liver biopsies: the first at the time of laparoscopic adjustable gastric band placement and the second after weight loss. The initial biopsies revealed NASH in 23 patients and steatosis in 12 patients. Follow-up biopsies were taken at 25.6 ± 10 months (range, 9-51 months) after band placement. There were significant major improvements in lobular steatosis, necroinflammatory changes and fibrosis at the second biopsy. Portal abnormalities remained unchanged. Eighteen patients had an initial fibrosis score of 2 or more, while only three patients showed this at the follow-up.

Jaskiewicz et al.^145^ conducted a study on 10 individuals who underwent a wedge biopsy during gastroplasty and had a new repeat biopsy eight months later. They observed significant improvements of the degenerative and inflammatory hepatic lesions in the repeat biopsies and liver function readings, eight months after the surgery.

In a prospective study conducted by Phillips et al.,^126^ proton magnetic resonance spectroscopy and magnetic resonance imaging were used to estimate the quantities of lipids contained within the liver and abdominal subcutaneous and visceral compartments of 29 obese individuals, before gastric banding and three months afterwards. Significant reductions in body weight, abdominal fat and liver fat were observed three months after surgery. Changes in liver fat content were more closely associated with changes in serum gamma-glutamyl transferase than with changes in waist circumference.

Gastaldelli et al.^127^ conducted a prospective study that evaluated 159 subjects with morbid obesity following laparoscopic adjustable gastric bypass. They observed that one year after gastric bypass, the patients’ glucose tolerance, liver function tests and IR had improved; ferritin had not changed significantly but was still correlated with IR. The authors concluded that ferritin might be an additional useful marker for cases of hepatic IR of greater severity.

A prospective study by Moschen et al.,^146^ in which 30 individuals who underwent gastric bypass were enrolled, showed that the surgery improved IR, abnormal liver function tests and liver histology. Pronounced weight loss after 6 and 12 months was accompanied by significant increases in serum adiponectin levels, whereas both leptin and visfatin levels decreased. Serum levels of resistin increased after 6 months but fell below baseline values after 12 months. These results suggest that weight loss after gastric bypass surgery improves the adipocytokine milieu towards a more anti-inflammatory direction, both systemically and in the liver.

In a retrospective study that enrolled 16 individuals, Swierczynski et al.^147^ observed significant improvements in several laboratory parameters, including serum phenylalanine, ALT, lipid concentrations and IR. A strong positive correlation between serum phenylalanine and serum ALT concentrations might suggest that the deterioration of liver function observed in obese patients contributes towards decreased phenylalanine metabolism and consequently towards increased serum phenylalanine concentration.

A prospective study was conducted by Frige et al.^148^ with the aim of comparing the effects of gastric bypass and biliointestinal bypass (BIB) on glucose and lipid metabolism in NAFLD. There were 24 individuals in the gastric bypass group and 12 in the BIB group, and a significantly greater decrease in liver enzymes (ALT) was observed in the gastric bypass patients than in the BIB group.

#### Sleeve gastrectomy

In an analysis on prospective data gathered from 236 individuals, Karcz et al.^128^ observed significant improvements in AST, ALT, triglycerides and high-density lipoprotein (HDL) levels one and three years after SG. NASH patients showed significant losses of body weight and amelioration of NASH status.

A retrospective study was conducted by Algooneh et al.^129^ on 84 patients diagnosed with NAFLD prior to undergoing SG. The diagnosis of NAFLD was achieved based on ultrasonographic imaging. A total of 47 patients (56%) showed complete resolution of NAFLD postoperatively. Multivariate analysis showed that there was significant resolution of NAFLD among the patients who achieved > 50% excess weight loss, after controlling for age and sex.

A prospective study conducted by Coupaye et al.,^149^ primarily to compare the nutritional effects of SG with those of RYGB, with 30 individuals in each group, also observed that transaminase levels were significantly lower after RYGB than after SG. This suggested that alterations in liver metabolism might affect synthesis or catabolism of some circulating lipids and proteins after RYGB.

Billeter et al.^150^ conducted a retrospective matched study on a prospective database, to compare the effects of RYGB and SG on 34 individuals with morbid obesity. Both procedures significantly lowered ALT and aspartate aminotransferase (AST) after 12 months, but SG improved both liver function tests significantly better than RYGB did. In contrast to RYGB, SG normalized elevated ALT levels completely. In a study comparing 30 individuals who underwent paired liver biopsies during surgery and six months afterwards, Praveen-Raj et al.^151^ reported that the individuals who underwent SG presented non-significant greater improvement of liver histology than those who underwent RYGB.

### Biliopancreatic diversions

#### Scopinaro operation

In a retrospective analysis on 99 consecutive individuals who underwent surgery, Papadia et al.^130^ noticed that AST levels at two months were significantly higher and then were significantly lower at 12 months. Hepatocellular necrosis in this series peaked at two months and decreased thereafter. The weight loss at two months, preoperative body weight, glucose levels and hepatic histology seemed to be of help in identifying patients at increased risk of acute liver damage, thus prompting the need for enhanced surveillance.

Ferrer Márquez et al.^131^ conducted a prospective study on 76 obese individuals who underwent biopsy during surgery and 12-24 months afterwards. They observed that there was a significant decrease in overall NAFLD, with decreased inflammation and fibrosis. No cases of liver failure were observed.

#### Duodenal switch

In an analysis on 104 individuals who underwent the duodenal switch procedure and required further reoperation, Kral et al.^132^ observed that severe fibrosis (grade 3-5) decreased in 28 whereas mild fibrosis (grade 1-2) appeared in 42. Increased fibrosis was related to low-normal serum albumin, uncontrolled diarrhea, low intake of alcohol and menopausal status. Fibrosis and inflammation significantly decreased over time. The 11 patients with cirrhosis exhibited decreases in fibrosis, from a mean of grade 5 to grade 3, as well as reduced levels of inflammation, Mallory bodies and glycogenated nuclei. Seven patients presented disappearance of nodules and fibrous bridging, while two showed regression.

Keshishian et al.^133^ conducted a retrospective analysis on 697 individuals who underwent laboratory tests 6, 12 and 18 months after surgery. All of them underwent liver biopsies during duodenal switch surgery and 78 individuals underwent a second surgical procedure at least six months after the primary duodenal switch. Transient elevation of liver enzymes at six months was observed; this was seen to have normalized at 12 and 18 months. A three-grade progressive improvement in the severity of NASH and a 60% improvement in hepatic steatosis were attained three years after the duodenal switch operation.

In a study on different procedures, Weiner^152^ analyzed 16 individuals who underwent biopsy during duodenal switch and in a later reoperation. Complete recovery from NAFLD was observed, and it was concluded that obesity surgery improved steatosis, necroinflammatory activity and hepatic fibrosis in patients with morbid obesity and NASH.

Johansson et al.^153^ conducted a prospective cohort study on 10 individuals who underwent duodenal switch and 21 cases of RYGB. Patients with morbid obesity treated by means of RYGB or duodenal switch showed sustained reductions in liver enzyme levels.

In a retrospective cohort study evaluating the influence of duodenal switch surgery on liver enzyme levels over a four-year period, among morbidly obese patients with normal aminotransferase and high baseline aminotransferase levels, Aller et al.^154^ reported that significant decreases in ALT and AST activity levels occurred. The baseline percentages of high aminotransferase levels and the percentage of ALT/AST ratios that were < 1 were also found to be significantly lower at the one, two, three and four-year follow-ups in both groups.

### Systematic reviews and synthesis

A meta-analysis conducted by Mummadi et al.,^157^ in which the surgical techniques analyzed were mixed, revealed that steatosis, steatohepatitis and fibrosis appeared to improve or become completely resolved in the majority of patients after bariatric surgery-induced weight loss. These authors emphasized the limitations of their study, regarding mainly the vast heterogeneity of the overall designs and expected outcomes of the studies included. Another meta-analysis, conducted by Bower et al.,^156^ revealed that bariatric surgery was associated with a significant reduction in the weighted incidence of a number of histological features of NAFLD, including steatosis (50.2%), fibrosis (11.9%), hepatocyte ballooning (67.7%) and lobular inflammation (50.7%); surgery was also associated with a reduction in liver enzyme levels. These authors emphasized that there was high heterogeneity among the methodologies and results, a factor that may have limited their analysis of these findings. A Cochrane review^157^ that specifically addressed the lack of randomized clinical trials and high heterogeneity of the studies available acknowledged that the improvements regarding steatosis and inflammation seemed to be clear. However, it was concluded that the quality of the data available did not allow the authors to draw any unbiased conclusion relating to bariatric surgery for treating NASH. Despite the limitations of these studies, they represent the best level of evidence currently available on this topic.

There are some major concerns regarding research on the effects of bariatric surgery for treating NAFLD that need to be emphasized. Firstly, as is common among surgical studies, there is a lack of randomized controlled studies; in fact, the majority of studies are retrospective and non-controlled, and these aspects may produce data of poor quality. Secondly, and specifically regarding studies on NAFLD, there is no standardized measurement for the outcomes evaluated: some studies have evaluated liver biopsy specimens, while others have compared data from imaging studies or noninvasive methods for assessing liver disease. Thirdly, optimal evaluation of NAFLD is only possible by means of liver biopsies. These can easily and safely be carried out at the time of the primary surgery, but this procedure is not widely available later on, given that it is not risk-free and therefore performing it in individuals who do not require surgical interventions constitutes an ethical concern. This leads to great heterogeneity of outcomes that also cannot be ignored.

Despite the lack of controlled and randomized studies, there is a clear trend towards better results with regard to NAFLD outcomes among obese individuals who undergo surgery. It is also clear that the staging of NAFLD at the time of surgery is an important predictor of success, since it is noticeable that the rates of resolution for simple steatosis are much higher than those observed for steatohepatitis and especially for fibrosis.

### Mechanisms underlying post-surgical improvements in NAFLD

The exact mechanisms that lead to improvement in NAFLD following bariatric surgery are probably strongly related to the metabolic changes that are involved in amelioration of metabolic syndrome (MetS). These changes occur early after the procedures, during a phase when no significant weight loss has yet been achieved. It is clear that while weight loss plays an important role in controlling metabolic abnormalities, this effect appears to be more significant over the long term.^158^^,^^159^^,^^160^ Furthermore, there is evidence that excess weight is not the main pathophysiological feature relating to NAFLD.^158^

The causes of early improvement of MetS after bariatric surgery are complex and relate to changes on the entero-insular axis mediated by gastrointestinal hormones called incretins.^160^^,^^161^^,^^162^ The anatomical changes caused by the surgical procedures, particularly duodenal exclusion and overflow of nutrients to the distal small bowel, lead to release of incretins, especially glucagon-like peptide 1 (GLP-1) and gastrointestinal insulinotropic polypeptide (GIP). These increase the production and release of insulin, improve peripheral insulin sensitivity and favor an anorexic state. Moreover, it appears that surgery enhances adipokine metabolism, which probably relates to increasing insulin sensitivity and promotes an anti-inflammatory effect by means of immunomodulation.^160^ Recent research has indicated that surgery leads to changes in the gut microbiota and bile acid circulation that may be beneficial regarding MetS and NAFLD.^158^^,^^159^^,^^160^^,^^161^^,^^162^

Furthermore, there is a decrease in portal influx of free fatty acids following surgery, which may be related both to feeding restrictions and to metabolic changes in visceral insulin sensitivity.^161^^,^^162^

### International Federation for the Surgery of Obesity and Metabolic Disorders (IFSO) position statement

In the most recent IFSO consensus statement,^163^ it is stated that bariatric surgery leads to reversal or significant improvement of NAFLD and NASH. Since the vast majority of the studies that led to this conclusion were non-randomized, non-controlled and observational cohort studies, this postulate constitutes a grade B recommendation, with evidence level 2. The IFSO also concluded that, despite the solid evidence available to date, further research, especially in randomized controlled settings, is necessary in order to reach ultimate conclusions. The high prevalence of NAFLD among the bariatric population also needs to be considered, as a significant factor.^17^^,^^164^^,^^165^ Hence, taking into account the overall impact of bariatric surgery on obesity and obesity-related comorbidities, such as NAFLD, this treatment option should be offered to the group of individuals who fulfill the current criteria of indications, as a potentially effective therapy.

#### Bariatric surgery in cirrhotic patients

Despite the increasing prevalence of obesity among the population with liver cirrhosis, along with the general epidemic of obesity, the number of studies on this topic is still limited. The main factors that need to be taken into account whenever a candidate for bariatric surgery is cirrhotic are the presence of portal hypertension and abnormalities of hepatocytic function. Regarding obese individuals who are also candidates for liver transplantation, the choice of technique and optimal timing for performing bariatric surgery are also significant issues that are not fully addressed in the current literature.

Among individuals with severe cirrhosis, surgical morbidity and mortality are significantly higher than what is observed in the general obese population. A retrospective study by Mosko et al.^166^ that analyzed a national United States database found significantly higher mortality rates among cirrhotic individuals, both in those who were clinically compensated (0.9%) and in those who were not (16.3%), than among non-cirrhotic individuals (0.3%). Moreover, in low-volume centers, mortality reached 41% among non-compensated cirrhotic individuals. Dallal et al.^167^ conducted a study that enrolled individuals with an incidental intraoperative diagnosis of liver cirrhosis, determined during bariatric surgery. They found that, among compensated cirrhotic individuals, RYGB presented a mortality rate similar to what was observed in the general population. However, it was also observed that episodes of transient renal failure, longer operation times and greater amounts of intraoperative bleeding and need for transfusion were more common within this group than in the general obese population. In a meta-analysis, Lazzati et al.^168^ observed a mean excess weight loss of 66% among morbidly obese cirrhotic individuals over a two-year period, which was comparable to the general population. SG was the surgical procedure most often performed, and the perioperative mortality rate was comparable to what was observed in the general population. However, morbidity (especially due to the need for reoperations) and mortality during the first year following surgery were significantly higher. The heterogeneity of the studies and the small samples analyzed were considered to be major limitations of their review, thus not allowing further conclusions.

Among candidates for liver transplantation, the choice of technique is of major importance, because of two factors: potential impairment of the absorption of immunosuppressive drugs and the possibility of endoscopic access to the biliary tract. Currently, there are no studies analyzing the pharmacokinetics of immunosuppressive drugs in liver-transplanted individuals who underwent bariatric surgery, but among kidney transplantation recipients who underwent RYGB, there are reports of the need for higher dosages of tacrolimus, sirolimus, mycophenolate sodium and cyclosporine.^169^ Regarding access to the biliary tract, post-transplantation biliary strictures are common, occurring in up to 17% of the cases.^170^ Taking these situations into consideration, SG appears to be the most appropriate technique for this group of individuals, since it does not affect drug absorption and enables endoscopic access to the biliary tract.

The optimal timing for performing bariatric surgery in individuals with cirrhosis who are also candidates for liver transplantation is yet to be determined. This surgery may be performed before, after or even during the transplantation.^171^ One significant concern is the potential impact of obesity on the outcomes from liver transplantation. Recently, it was observed that surgical mortality, two-year survival and graft viability were similar in obese and lean individuals. Perioperative morbidity was slightly higher.^172^^,^^173^ Because of the higher morbidity and mortality observed among non-compensated liver transplantation candidates, the pre-transplantation approach presents clear limitations. Bariatric procedures performed concomitantly with the transplantation have been reported; this approach has the potential to minimize the number of surgical procedures in high-risk individuals. However, it also requires concomitant availability of both surgical teams and generates the risk of combining the complications of both procedures.^174^ Based on this latter finding, although there is no consensus yet, the approach that is best accepted is to perform the transplantation first and the bariatric surgery later on, among non-compensated cirrhotic individuals or among those with moderate to severe portal hypertension. According to the current literature, the morbidity is significantly higher, but the mortality is comparable.^175^^,^^176^

#### Current situation and future perspectives

NAFLD is becoming a public health concern worldwide today, and its prevalence is expected to grow even further in the near future. There is enough evidence to suggest that bariatric surgery should be considered to be the optimal treatment option for NAFLD by choice, and no longer only by chance.^177^ It is expected that NAFLD may become the major reason for living-donor transplantation in the United States by 2030.^178^

Although bariatric surgery is currently recognized as the standard treatment option for morbid obesity and its related comorbidities, the number of operations performed is far from sufficient to meet the need for intervention. In fact, less than 500,000 bariatric surgery procedures are performed worldwide every year today. This number represents less than 0.1% of the entire supposedly obese population.^179^^,^^180^^,^^181^ Moreover, there are large numbers of subjects who do not fulfill the current indications for surgery but present harmful abnormalities relating to fat and glucose metabolism that can lead to and aggravate liver disease.

Hence, the most practical and effective action that can be implemented in relation to this entire disease chain is prevention. Encouragement towards practicing physical activity and following healthy low-fat/low-calorie diets needs to be extensively included in educational and community programs as early in life as possible, since obesity has become a pediatric issue too.^179^^,^^180^ Continuing education for healthcare professionals regarding liver disease and the methods for detecting, preventing and treating it should be mandatory. Screening of populations at high risk, in order to provide early diagnosis and staging of NAFLD, may also be carried out by means of simple laboratory tests and ultrasound scans. Early detection of MetS and diabetes and prompt management of these illnesses remain significant ways for preventing evolution to liver disease and its ominous severe forms.^180^^,^^181^

Regarding the role of bariatric surgery in relation to the clinical course of NAFLD, many questions remain to be answered. Since there is a lack of randomized controlled trials, no ultimate conclusions can yet be provided. It is clear that the overall impact of bariatric surgery on NAFLD is positive, but the optimal surgical technique remains to be determined, as do the longer-term effects and the ways of maintaining the benefits achieved. Since there is also concern regarding the onset of persistent IR or even re-emergence of IR and T2DM^182^^,^^183^ among individuals who initially presented complete reversal, further research is needed in order to determine the influence of this event on liver histology. Another longer-term concern is the prevalence of weight regain, which may also have a significant effect on NAFLD.

### Statement of human and animal rights

All procedures performed in the studies evaluated involving human participants were in accordance with the ethical standards of the institutional and/or national research committee and with the 1964 Helsinki declaration and its later amendments or comparable ethical standards.

## CONCLUSION

The currently available evidence shows that bariatric surgery may become the treatment of choice for individuals with NAFLD. It is important to emphasize that further research, especially by means of randomized controlled trials enrolling larger cohorts of individuals, is necessary in order to determine the optimal procedure for this group of subjects, as well as whether only individuals with morbid obesity might benefit from the effects of metabolic surgery, since there are vast numbers of lean and overweight individuals who also present NAFLD.
